# Comparison of Risk Stratification Tools for Atherosclerotic Cardiovascular Disease and Cardiovascular–Kidney–Metabolic Syndrome in Primary Care

**DOI:** 10.3390/medsci13040240

**Published:** 2025-10-23

**Authors:** Victor Hugo Vázquez Martínez, Humberto Martínez Bautista, Patricia Muñoz Villegas, Jesús III Loera Morales, María del Rosario Padilla Salazar

**Affiliations:** 1 Instituto Mexicano del Seguro Social, Unidad de Medicina Familiar No. 33 de Reynosa, Tamaulipas 88620, Mexico; jesus.loeram@imss.gob.mx (J.I.L.M.);; 2 Centro de Investigación en Matemáticas, A.C. (CIMAT), Unidad Aguascalientes, Aguascalientes 20200, Mexico; humberto.martinez@cimat.mx (H.M.B.); patricia.munoz@cimat.mx (P.M.V.)

**Keywords:** chronic non-communicable diseases, atherosclerotic cardiovascular disease, cardiovascular–kidney–metabolic syndrome, ordinal logistic regression model, CKM prevention

## Abstract

**Background/Objectives**: Cardiovascular disease is the leading cause of death in Mexico; this is due to the high prevalence of chronic non-communicable diseases (NCDs), including obesity, type 2 diabetes mellitus (T2DM), systemic arterial hypertension (SAH), cardiovascular disease, chronic kidney disease (CKD), and dyslipidemia. Primary care physicians require a classification tool that enables them to gain a broader understanding of their patients’ risks, thereby allowing them to make more informed clinical decisions. This study compared risk stratification for atherosclerotic cardiovascular disease (ASCVD) and Cardiovascular–Kidney–Metabolic (CKM) syndrome in a primary care setting in Mexico. **Methods**: An observational, descriptive, cross-sectional study analyzed 500 patients with T2DM, SAH, dyslipidemia, and/or CKD. Two ordinal logistic regression models were developed using a Chi-square test, Kruskal–Wallis test, and tetrachoric, polychoric, polyserial, and Pearson correlations. **Results**: Associations were found between ASCVD risk and factors like sex, age, and T2DM; for CKM syndrome, the associations were with age, T2DM, and dyslipidemia. Interestingly, 22% of advanced CKM patients had a low ASCVD risk. Alcohol consumption showed a strong positive relationship (42%) with CKM stages, while there was a negative relationship (33%) with the glomerular filtration rate. **Conclusions**: The ASCVD risk classification effectively identifies cardiac conditions, but the CKM syndrome score provides a broader assessment of comorbidities at earlier stages. Key factors like age, hypertension, T2DM, and smoking are crucial for cardiovascular risk but less so for CKM syndrome, highlighting the need for a broader stratification of risk.

## 1. Introduction

Ischemic Heart Disease has a global incidence rate of 1655 for 100,000 people and a disability rate of 2228 for 100,000. The regions most affected globally are those with low-to-medium levels of development [1,2]. In the Americas, some countries reduced cardiovascular mortality; however, an increase of 0.6% has been observed in countries such as Mexico [3]. The increase in cardiovascular events in Mexico is primarily due to the coexistence of various risk factors, including a 12.2% prevalence of type 2 diabetes mellitus (T2DM), 47.8% prevalence of systemic arterial hypertension (SAH), and 9% prevalence of dyslipidemia [4,5,6].

Due to Mexico’s high cardiovascular mortality rate, primary care physicians consistently utilize the cardiovascular risk assessment from the American College of Cardiology and the American Heart Association (AHA) Task Force, which considers risk factors such as age, sex, race, blood pressure, low-density lipoprotein cholesterol (LDL-C), high-density lipoprotein cholesterol (HDL-C), and total cholesterol. This assessment enables primary care physicians to implement preventive measures based on individual risk [7]. However, the AHA has recently stated that a new approach is needed to unify heart disease, kidney disease, diabetes, and obesity under a single definition known as cardiovascular–kidney–metabolic (CKM) Syndrome. CKM syndrome is a “systemic disorder characterized by pathophysiological interactions among metabolic risk factors, chronic kidney disease, and the cardiovascular system leading to multiorgan dysfunction and a high rate of adverse cardiovascular outcomes”. This CKM classification consists of five stages, enabling primary care physicians to identify progressive pathophysiology and the increasing absolute risk of cardiovascular disease. The factors considered for risk stratification include a body mass index (BMI) of 25 kg/m^2^ or greater, abdominal obesity (waist circumference ≥88 cm in women and ≥102 cm in men), hypertriglyceridemia, hypertension, diabetes, chronic kidney disease (CKD), and cardiac biomarkers [8,9,10]. Furthermore, this approach aims to screen, classify, and evaluate CKM syndrome early in both childhood and adulthood, while also promoting an interdisciplinary strategy for prevention and management [11,12]. In consensus, the treatment of CKM syndrome should involve a holistic approach focused on prevention, detection, and management to minimize long-term morbidity and mortality [13]. The ASCVD risk stratification has been used consistently for more than a decade; however, a broader approach is needed due to the simultaneous presentation of various diseases in patients [14]. The objective of the present study was to compare risk stratification for atherosclerotic cardiovascular disease (ASCVD) and cardiovascular–kidney–metabolic (CKM) syndrome in primary care settings from a public health institution in Mexico.

## 2. Materials and Methods

### 2.1. Participants

This observational, descriptive, cross-sectional study was conducted at Family Medicine Unit No. 33 in Reynosa, Tamaulipas, Mexico, under the auspices of the Mexican Institute of Social Security (IMSS). The unit comprises 54 primary healthcare medical professionals who provide general consultations to 208,000 beneficiaries. The study, conducted from January 2023 to December 2024, received authorization from the Research Ethics Committee (No. 28028) and the Local Health Research Committee (registration number R-2023-2802-013) before data collection. During the study period, a total of 1376 patients were referred for the first time from the primary care clinic to the Cardiology Department of the General Hospital Number 15 of the IMSS in the specified city, for patient evaluation. From this population, we estimated a proportion sample size, considering a 50% prevalence of hypertension, which is the most common cardiovascular risk factor. After this calculation, the sample size was increased by 30% to account for possible loss to follow-up, those who retired due to adverse events, or any other factors that compromised the validity of the findings. A total of 500 participants who provided and signed informed consent voluntarily were included; male and female, aged 18 years and older, who had been diagnosed with T2DM, SAH, atherosclerotic syndrome, dyslipidemia, and/or CKD. The exclusion criteria included hospitalization for cardiovascular reasons within six months before data collection, as patients continued to be treated by the cardiologist department during this period, as well as incomplete or illegible surveys.

### 2.2. Data Collection

Sociodemographic, clinical, and laboratory data for participants who met the inclusion criteria were obtained from the family medicine information system and the electronic medical records of the referral hospital. Data collection was conducted using a structured questionnaire that encompassed various anthropometric variables, including weight, height, and BMI, as well as primary and secondary referring diagnoses, vital signs, and the medical history of SAH, T2DM, and CKD. Laboratory results obtained within the previous six months were included: glycated hemoglobin (HbA1c), glucose, urea, creatinine, triglycerides, total cholesterol, LDL-C, HDL-C, and glomerular filtration rate (GFR). Information on aspirin and atorvastatin use, smoking, and self-reported alcohol consumption habits was also collected.

The assessment of CKM syndrome was conducted in accordance with the AHA criteria. The stages are defined as follows: Stage 0: Absence of risk factors; Stage 1: Presence of excessive or dysfunctional adiposity; Stage 2: Detection of metabolic risk factors alongside CKD; Stage 3: Indications of subclinical cardiovascular disease; Stage 4a: Clinical cardiovascular disease without the presence of kidney failure; and Stage 4b: Clinical cardiovascular disease with accompanying kidney failure. This classification offers a comprehensive framework for understanding the progression of CKM syndrome, based on both clinical and metabolic indicators [8,9]. In addition, the ASCVD Risk Estimator Plus, developed by the American College of Cardiology, was used as a classifier. This tool takes into account several factors: current age, sex, race, systolic blood pressure, HDL cholesterol, LDL cholesterol, diabetes history, smoking status (current, former, or never smoker), treatment for hypertension, and whether the patient is on statin or aspirin therapy. After gathering this information, the ten-year risk for ASCVD is categorized as follows: low risk (less than 5%), moderate or borderline risk (5% to 7.4%), intermediate (7.5% to 19.9%), and high risk (greater than 20%) [15].

Laboratory tests were conducted in the Family Medicine Unit No. 33 using an automated, calibrated device known as Atellica^®^ Solutions, developed by Siemens in Erlangen, Germany. Laboratory parameters were standardized with Synchorinzing Standard Operating Procedures to ensure consistency and efficiency between the primary care clinic and the Hospital. Both institutions share a digital platform called Modulab Laboratory© Version 4.0.01 (Werfen, Germany), which allows health professionals to access it. Furthermore, the provider provides training in the validation and management of results.

### 2.3. Statistical Analysis

To examine the global distribution of the classifiers, ASCVD risk, and CKM syndrome, we conducted both univariate and bivariate descriptive analyses. For variables with categorical scales, we presented totals, frequencies, and percentages. For quantitative variables, we calculated the mean and standard deviation. Additionally, the Chi-square test was employed to analyze the association between the two classifiers and each of the factors. To compare the distribution of the two classifiers across continuous variables, the Kruskal-Wallis (K-W) test was utilized [16], which is robust against non-normality and does not require the assumption of homogeneity of variances.

**Figure 1:**
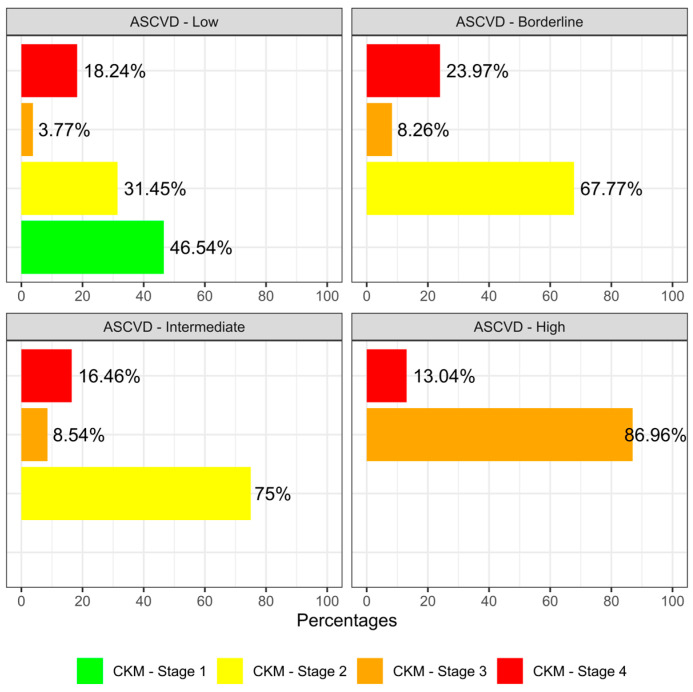
Distribution of the CKM syndrome in the different stages of ASCVD risk classification. Bars show frequency (%).

**Figure 2:**
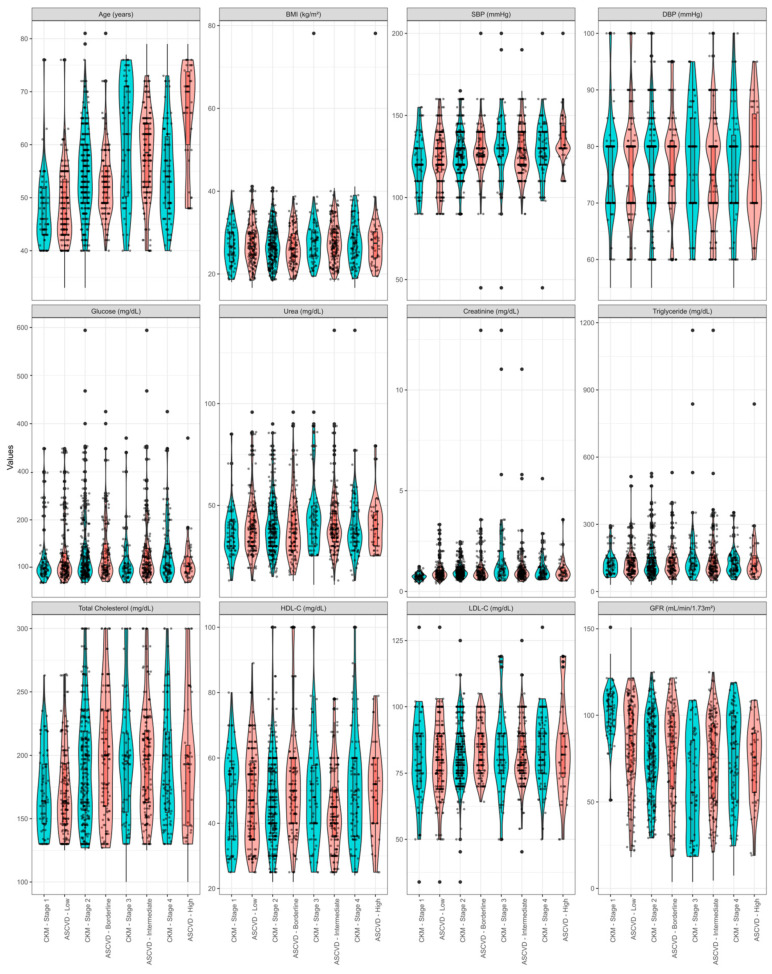
Distributional analysis between homologous levels of ASCVD risk and CKM syndrome. The quadrants illustrate the distribution of data for each variable analyzed in each classifier. Green violin plots corresponding to ASCVD and red violin plots refer to CKM syndrome.

The graphical analysis in Figure 1 contrasts patient classification by ASCVD risk with CKM syndrome. Figure 2 displays the central distribution (median), dispersion (interquartile range), and outliers of the quantitative variables under study.

The relationship between ASCVD risk, CKM syndrome, and various influencing factors was evaluated by calculating a polychoric correlation matrix. This approach was appropriate given the characteristics of the variables involved in the study. For dichotomous variables, which have two categories, the tetrachoric correlation—a specific case of the polychoric correlation—was used. For ordinal variables, the polychoric correlation was computed. These correlation types are preferred because ASCVD risk and CKM syndrome are measured on an ordinal scale, making this method the most suitable for these measurements. The correlation results indicate the degree, strength, and direction of the relationship between the two factors. A positive correlation means that both factors move in the same direction; as one factor increases, the other also increases in value. In contrast, a negative correlation indicates that the factors move in opposite directions: as one factor increases, the other decreases, or vice versa.

When examining the relationship between a continuous variable and a categorical variable, the polyserial correlation was used. Finally, to assess the correlation between two continuous variables, the Pearson correlation was used [17].

Two ordinal logistic regression (OLR) models were developed based on univariate, bivariate, graphical, and correlational analyses [18]. The process began by fitting a statistically valid model for ASCVD risk, ensuring parsimony through the use of the Akaike Information Criterion (AIC) and the Bayesian Information Criterion (BIC). Calibration was assessed using the Hosmer–Lemeshow test for ORL models, and adequate model specification was verified with the link test [19,20]. The CKM syndrome model was then refined by incorporating the covariates identified in the earlier stage. We explored a comprehensive stepwise modeling approach with automated variable selection. This method enabled us to identify confounding, modifying, and controlling factors and to adjust the model formulation accordingly. Statins, antihypertensives, and antidiabetic drugs were not included in the model because their prescription may underestimate cardiovascular and cardio-renal metabolic syndrome.

Finally, a significance level of 5% (α = 0.05) was utilized for all analyses, except for the correlational analysis, where a significance level of 10% (α = 0.10) was applied. Statistical analyses were conducted using Stata version 19.5, serial number: 401909322840 (StataCorp LLC, TX, USA, 2025) and R version 4.5.0 (The R Foundation for Statistical Computing; http://www.R-project.org, accessed on 1 June 2025).

## 3. Results

The participant sex distribution was 44% men and 56% women, with age and nutritional status categories varying from 27% to 39% for each variable. High prevalence rates were recorded for several health concerns: 85% for SAH, 57% for T2DM, and 77% for alcohol consumption habits. A bivariate chi-square analysis revealed a statistically significant association (*p* < 0.05) between the risk level for ASCVD and the factors of sex, age group, and T2DM. Additionally, for CKM syndrome, associations were found with age, T2DM, and dyslipidemia, the latter showing a *p*-value of 0.069 (see Table 1).

When comparing patient stratification using the ASCVD and CKM classifiers, it was noted that 22% of patients with CKM in advanced stages were classified as having a low ASCVD risk, i.e., that if only the ASCVD risk classifier is used, one-fifth of patients who are at high risk (stages 3 and 4) for CKM syndrome would go unrecognized, resulting in missed opportunities for appropriate health interventions based on their actual risk levels. Patients identified as being at high risk for ASCVD were classified during stages 3 and 4 of CKD syndrome. Suggesting that the classifiers are consistent at high levels of these indices; however, they do not show the same level of consistency at low levels of ASCVD (see Figure 1).

Table 2 presents the total and classifier estimates of centrality, dispersion, and the distributional comparison using the mean, standard deviation, and the K-W test. The K-W test revealed statistically significant differences for age, systolic blood pressure (SBP), total cholesterol, and GFR in both ASCVD risk and CKM syndrome. Indicating that there are differences among at least two categories for each classifier. Additionally, the cholesterol showed statistical significance in the K-W test for ASCVD risk, while in the case of CKM syndrome, significant findings were noted for HbA1c, glucose, and creatinine levels.

Figure 2 illustrates a comparison of quantitative demographic, anthropometric, and clinical characteristics, enabling the stratification of patients based on ASCVD risk and CKM syndrome. Notably, there is greater variability in age among patients classified as CKM syndrome stage 4 compared to those identified as having high ASCVD risk. Additionally, patients with CKM syndrome stage 4 tend to be diagnosed at a younger age. At a younger age than typically observed for the diagnosis of high ASCVD risk, several factors showed elevated levels, including glucose, urea, creatinine, and triglycerides. These factors consistently presented high levels across nearly all evaluations (see the second row of Figure 2) for both ASCVD risk and CKM syndrome. The poor glycemic control among patients may account for these findings. Additionally, factors such as nutritional status, as indicated by BMI, diastolic blood pressure (DBP), total cholesterol, HDL-C, and LDL-C, demonstrated consistent behavior across most classification levels.

The results of the correlational analysis offer a detailed insight into the strength and direction—either positive or negative—of the relationship between the two classifiers: ASCVD risk and CKM syndrome. The analysis revealed a positive correlation of approximately 40%. Additionally, it examined the interrelationships among all the factors studied. A statistical significance level of 10% (*p* < 0.10) was adopted to identify a broader range of explanatory variables.

**Figure 3:**
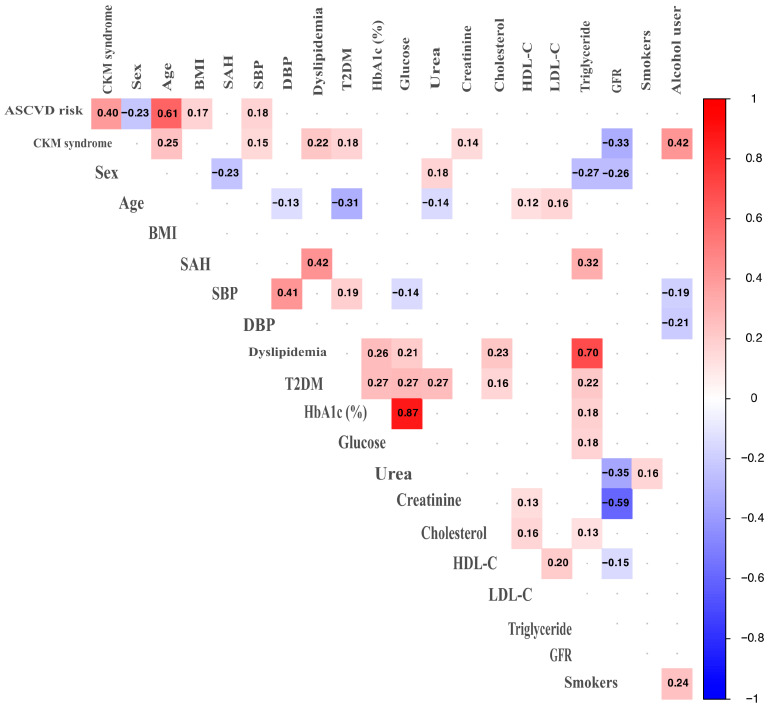
Polychoric correlation analysis between factors and classifiers for ASCVD risk and CKM syndrome. Only significant correlations are shown (*p* < 0.10).

ASCVD risk levels and age have a positive relationship of over 60%. For sex, a negative relationship was found with a value of 23%. SBP was positively related at 18%, and finally, BMI had a positive relationship with a value of 17%. CKM syndrome stages have a strong positive relationship (42%) with alcohol consumption habits, a negative relationship of 33% with GFR, and are positively related to age at 25%. The impact of age is lower for CKM syndrome, unlike ASCVD risk, dyslipidemias, and T2DM, which are positively associated with values of 22% and 18%, respectively. SBP has a positive effect with a value of 15%, similar to that obtained for ASCVD risk. Finally, a positive relationship was also found between CKM stages and creatinine levels in 14% of cases (see Figure 3).

There is a strong positive correlation between dyslipidemia and triglycerides, with a correlation coefficient of 0.70. Additionally, a significant relationship exists between creatinine levels and GFR, as indicated by a correlation coefficient of −0.59. A moderate correlation was observed between dyslipidemia and the presence of SAH, with a coefficient of 0.42. Furthermore, a medium correlation exists between triglycerides and SAH, with a correlation coefficient of 0.32, as well as between GFR and urea levels, with a correlation coefficient of −0.35. The remaining comparisons exhibited low correlations (see Figure 3).

**Figure 4:**
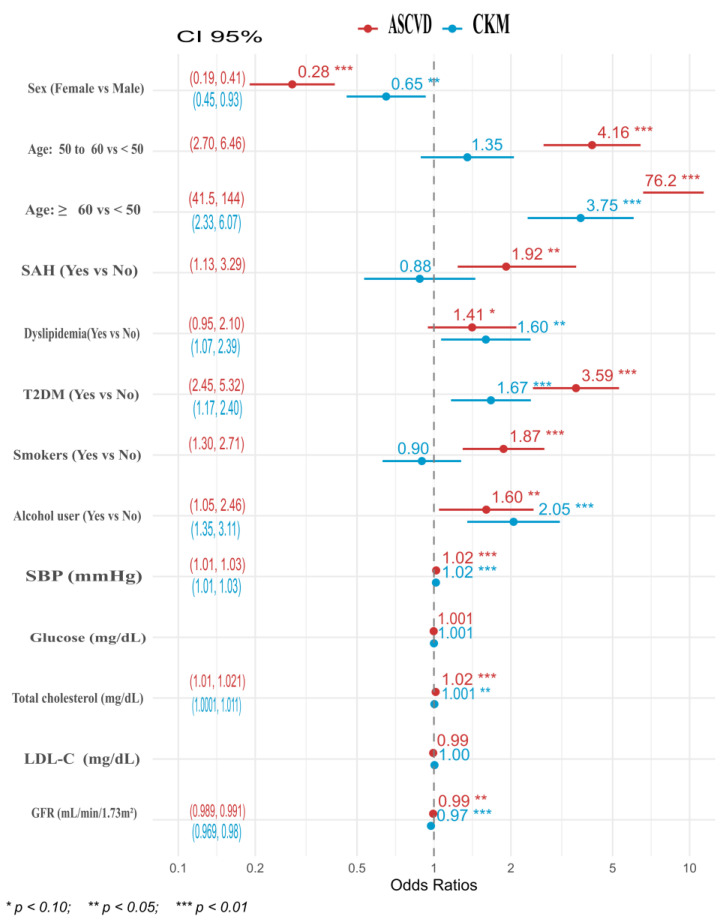
Ordinal logistic regression (ORL) models for ASCVD risk and CKM syndrome.

The adjusted results from the two ORL models enabled comparisons of risk factors related to ASCVD and CKD syndrome. For patients aged between 50 and 60 years, the risk for ASCVD was four times higher, while the risk for CKM syndrome was not significant. In adults over 60, the risk of ASCVD remained elevated, with a risk score of 3.75 compared to patients younger than 50. It indicates that advanced age is a significant determinant of cardiovascular disease severity. However, advanced age has a lower impact on the development of CKM syndrome, suggesting that there may be an opportunity to manage other metabolic factors that could enhance the quality of life for older individuals. The female sex showed protective behavior compared to the male sex. For ASCVD risk, it was 72%, which was higher (35%) compared to ASCVD syndrome (Figure 4).

High blood pressure (SAH) was a risk factor for ASCVD but not for CRM syndrome. Dyslipidemias showed a greater risk impact (almost 20%) for CKM syndrome compared to the ASCVD risk. T2DM was identified as a risk factor for both classifiers, with a greater effect for ASCVD (almost twofold). Smoking was identified as a risk factor for ASCVD, but not for CKM syndrome, and in patients with positive alcohol consumption habits, it had a greater impact on CKM syndrome. SBP, glucose, total cholesterol, LDL-C, and GFR showed no or minimal effects (between 1 and 3%) on the risk of ASCVD and CKM syndrome (see Figure 4).

## 4. Discussion

In this study involving 500 patients referred to the cardiology department of a secondary care hospital, we assessed the prevalence of T2DM, SAH, dyslipidemia, and alcohol consumption habits using ASCVD risk stratification and CKM syndrome staging. We found that 22% of the patients were classified as having a low ASCVD risk; however, when applying CKM staging, these patients were found to be in stages 3 and 4.

One challenge for primary care physicians is managing patients with multiple conditions, such as T2DM, SAH, dyslipidemia, CKD, and various social determinants that can lead to inaccurate ASCVD risk stratification. Lloyd-Jones DM et al. observed that this risk stratification tool can underestimate the risk for patients with low-income status and overestimate it for those with high-income status; this social variable may have contributed to our results [21]. However, we consider that the existing epidemiological profile in Mexico, characterized by a high percentage of obesity, dyslipidemia, T2DM, SAH, and CKD, requires a new approach with the use of a broader and more effective tool for cardiovascular risk stratification in primary care settings. The use of CKM syndrome staging enables primary and secondary care physicians to adopt an interdisciplinary and coordinated approach, facilitating timelier promotional, preventive, diagnostic, therapeutic, and rehabilitative actions to improve patients’ quality of life [22].

Health professionals need to consider the proportion of patients affected by CKM syndrome. Aggarwal, Ostrominski, and Vaduganathan reported that the prevalence of this syndrome among individuals over the age of 20 is 10.6% in stage 0, 25.9% in stage 1, 49% in stage 2, 5.4% in stage 3, and 9.2% in stage 4. This prevalence can be higher in an older population or with multiple morbidities like ours [23]. Cardiovascular disease is the leading cause of death among the Latino population in the United States. Research on the risk of ASCVD has yielded mixed results. Evidence suggests that the prevalence of risk factors may vary depending on the country of birth or cultural characteristics. Latinos in the US, regardless of their country of birth, are more often eligible for statin therapy for the prevention of ASCVD compared to non-Hispanic whites. This increased eligibility is primarily due to the high prevalence of diabetes, which affects 60% of United States-born Latinos and 68% of Latinos who were born outside the United States but currently reside there [24,25]. This prevalence aligns with our study, which found that 57% of patients were diagnosed with diabetes. T2DM is a recognized risk factor for cardiovascular disease and CKD. In the United States, racial and ethnic minority groups, including Hispanic/Latino individuals, non-Hispanic African Americans, and Southeast Asians, bear a disproportionate burden of T2DM and its associated complications [26]. Our findings indicate that T2DM is a significant risk factor for both ASCVD and cardiovascular risk classification metrics, with a notably greater impact on ASCVD, almost double that of other factors. These findings support the need for greater patient participation in preventive health behaviors, particularly among those at higher risk, such as individuals residing in areas with a high prevalence of non-communicable diseases, like Mexico [27].

It is important to note that in this study, the levels of total cholesterol, triglycerides, HDL, and LDL were in acceptable ranges; however, we must emphasize the importance of clinical risk factors and biomarkers for starting lipid-lowering therapy in patients with T2DM or other high-risk conditions that elevate the chances of a cardiovascular event [28,29,30].

LDL-C levels are essential factors in assessing the risk of cardiovascular disease. Numerous studies have shown a linear relationship between LDL-C levels and the occurrence of cardiovascular events. Therefore, managing LDL-C levels is a key therapeutic goal in clinical practice. Additionally, reducing LDL-C levels has been linked to a lower risk of ASCVD [31,32,33].

Furthermore, understanding the intensity of specific factors associated with cardiovascular risk and CKM syndrome in underserved communities is crucial due to the disproportionate presence of poverty, which limits access to healthy food, higher stress levels, and a high prevalence of smoking and alcohol use as coping measures and socialization. Factors such as age, sex, comorbidities like T2DM, SAH, smoking, alcohol consumption, and high cholesterol are helpful for an early stratification, so health professionals can implement preventive measures to avoid the progression of this disease [34,35,36].

Even though we found that smoking and alcohol consumption were risk factors for cardiovascular risk, we would like to establish that these variables were self-reported by the patient. We did not measure the quantity of alcohol or obtain the smoking index; this could contribute to the result. However, there is evidence that alcohol consumption is positively associated with 10-year CVD risk progression, and moderate drinking is correlated with increased mortality among males and females with CKM stage IV and II, respectively [37,38]. Furthermore, during the COVID-19 pandemic, there was a substantial increase in the prevalence of CKM syndrome, and almost half of the patients had at least one CKM syndrome component [39]. Therefore, a comprehensive approach is needed for an early stratification of CKM, especially among patients with multiple comorbidities in primary care settings.

Nevertheless, the ASCVD risk stratification tool will continue to be a convenient option in areas where resources are limited for performing laboratory tests necessary for risk stratification using the CKM scale. We faced this challenge due to the limited laboratory resources available for conducting stricter CKM stratification; therefore, we utilized the available clinical and physical variables to stratify CKM syndrome [40].

Our study does have some limitations. First of all, this is a cross-sectional design, and we cannot affirm that there was a longitudinal validation or proper patient follow-up from the early stages of ASCVD or CKM. The classifications were based on information obtained from the electronic medical record, which helped us determine the level of CKM syndrome for each included patient, as well as their ASCVD risk. However, some recommended methods for defining advanced CKM stages—such as cardiac biomarkers, coronary angiography, cardiac computed tomography, atrial fibrillation evaluation, and assessments for peripheral arterial disease—were not utilized. This absence of data may have led to an underestimation of stages 3 and 4 of CKM syndrome. In our country, these studies are not always routinely conducted in primary care. Therefore, the significance of our findings outweighs this limitation.

## 5. Conclusions

The ASCVD risk classification scale effectively stratifies individuals with cardiovascular disease (CVD) or cardiac conditions. However, the CKM syndrome score provides a more comprehensive assessment of other comorbidities at an earlier stage. Factors such as age, hypertension, T2DM, and smoking are critical determinants of cardiovascular risk; however, they play a limited role in identifying CKM syndrome. Instead, CKM syndrome more strongly emphasizes dyslipidemia and alcohol consumption as significant risk factors.

## Figures and Tables

**Table 1:** Distribution of categorical factors by ASCVD risk levels and CKM syndrome.

	Total	ASCVD Risk					CKM Syndrome				
	*n* = 500	Low	Borderline	Intermediate	High	*p*-Value ^1^	Stage 1	Stage 2	Stage 3	Stage 4	*p*-Value ^1^
		*n* = 160	*n* = 124	*n* = 170	*n* = 46		*n* = 74	*n* = 263	*n* = 72	*n* = 91	
Sex											
Male	222 (44)	53 (33)	64 (52)	80 (47)	25 (54)	0.004	34 (46)	114 (43)	32 (44)	42 (46)	0.959
Female	278 (56)	107 (67)	60 (4)	90 (53)	21 (46)		40 (54)	149 (57)	40 (56)	49 (54)	
Age											
<50 years	170 (34)	94 (59)	48 (39)	23 (14)	5 (11)	<0.001	47 (64)	73 (28)	17 (24)	33 (36)	<0.001
≥50 and <60 years	194 (39)	57 (36)	62 (50)	68 (40)	7 (15)		24 (32)	124 (47)	17 (24)	29 (32)	
≥60 years	136 (27)	9 (5.6)	14 (11)	79 (46)	34 (74)		3 (4.1)	66 (25)	38 (53)	29 (32)	
BMI											
Underweight to healthy weight	186 (37)	60 (38)	55 (44)	54 (32)	17 (37)	0.437	30 (41)	100 (38)	25 (35)	31 (34)	0.963
Overweight	174 (35)	58 (36)	40 (32)	61 (36)	15 (33)		23 (31)	93 (35)	25 (35)	33 (36)	
Obese	140 (28)	42 (26)	29 (23)	55 (32)	14 (30)		21 (28)	70 (27)	22 (31)	27 (30)	
SAH											
Yes	427 (85)	131 (82)	106 (85)	148 (87)	42 (91)	0.354	63 (85)	225 (86)	64 (89)	75 (82)	0.715
Dyslipidemia											
Yes	136 (27)	37 (23)	31 (25)	57 (34)	11 (24)	0.149	12 (16)	75 (29)	18 (25)	31 (34)	0.069
T2DM											
Yes	287 (57)	71 (44)	80 (65)	110 (65)	26 (57)	<0.001	30 (41)	158 (60)	43 (60)	56 (62)	0.017
Smokers											
Yes	228 (46)	72 (45)	54 (44)	80 (47)	22 (48)	0.925	36 (49)	124 (47)	30 (42)	38 (42)	0.677
Alcohol user											
Yes	114 (23)	36 (23)	33 (27)	33 (19)	12 (26)	0.489	13 (18)	55 (21)	22 (31)	24 (26)	0.187

Note: Values in frequency (%). ^1^ Chi-squared test for association among variables. Abbreviations: Atherosclerotic Cardiovascular Disease, ASCVD; body mass index, BMI; cardiovascular–kidney–metabolic, CKM; systemic arterial hypertension, SAH; type 2 diabetes mellitus, T2DM.

**Table 2:** Distribution and comparison of quantitative factors by intra-group levels of ASCVD risk and CKM syndrome.

	Total *n* = 500	ASCVD Risk					CKM Syndrome				
		Low	Borderline	Intermediate	High	*p*-Value ^1^	Stage 1	Stage 2	Stage 3	Stage 4	*p*-Value ^1^
		*n* = 160	*n* = 124	*n* = 170	*n* = 46		*n* = 74	*n* = 263	*n* = 72	*n* = 91	
Age (years)	54 ± 9	49 ± 7	53 ± 6	58 ± 8	66 ± 10	<0.001	48 ± 6	54 ± 8	61 ± 11	55 ± 9	<0.001
BMI (kg/m^2^)	27.2 ± 5.4	27.2 ± 5.0	26.4 ± 4.7	27.6 ± 5.0	28.2 ± 8.7	0.236	26.9 ± 5.2	26.8 ± 4.7	28.2 ± 7.4	27.8 ± 5.5	0.502
SBP (mmHg)	127 ± 17	124 ± 17	128 ± 18	128 ± 16	135 ± 16	<0.001	121 ± 16	127 ± 15	131 ± 21	129 ± 18	<0.001
DBP (mmHg)	77 ± 9	77 ± 10	78 ± 8	75 ± 9	77 ± 9	0.160	77 ± 9	76 ± 9	78 ± 9	77 ± 10	0.800
HbA1c (%)	6.9 ± 1.7	6.9 ± 1.7	7.1 ± 1.6	7.1 ± 1.8	6.4 ± 1.3	0.302	6.4 ± 1.5	7.2 ± 1.7	6.7 ± 1.6	6.9 ± 1.8	0.039
Glucose (mg/dL)	128 ± 65	127 ± 64	133 ± 64	130 ± 70	113 ± 47	0.318	118 ± 58	132 ± 69	116 ± 55	133 ± 64	0.029
Urea (mg/dL)	40 ± 15	40 ± 15	39 ± 16	40 ± 15	40 ± 12	0.600	36 ± 12	40 ± 13	45 ± 20	39 ± 16	0.057
Creatinine (mg/dL)	1.1 ± 0.9	0.9 ± 0.5	1.2 ± 1.2	1.1 ± 1.0	1.0 ± 0.5	0.104	0.7 ± 0.2	1.0 ± 0.4	1.8 ± 2.0	1.1 ± 0.7	<0.001
Total cholesterol (mg/dL)	191 ± 47	173 ± 35	202 ± 51	200 ± 48	190 ± 53	<0.001	172 ± 32	195 ± 49	198 ± 52	190 ± 45	0.003
HDL-C (mg/dL)	48 ± 15	48 ± 14	51 ± 17	44 ± 13	51 ± 13	<0.001	47 ± 13	47 ± 14	49 ± 15	50 ± 17	0.381
LDL-C (mg/dL)	82 ± 14	80 ± 17	84 ± 10	81 ± 11	84 ± 18	0.043	80 ± 19	81 ± 11	83 ± 16	83 ± 13	0.319
Triglyceride (mg/dL)	141 ± 102	130 ± 67	136 ± 76	153 ± 135	145 ± 120	0.859	122 ± 53	137 ± 79	185 ± 201	130 ± 59	0.151
GFR (mL/min/1.73 m^2^)	78 ± 27	84 ± 27	78 ± 28	74 ± 26	73 ± 22	0.002	104 ± 15	77 ± 23	58 ± 30	75 ± 28	<0.001

Notes: Value on mean ± standard deviation. ^1^ Kruskal–Wallis. Abbreviations: Atherosclerotic cardiovascular disease, ASCVD; cardiovascular–kidney–metabolic, CKM; diastolic blood pressure, DBP; glomerular filtration rate, GFR; glycated hemoglobin, HbA1c; high-density lipoprotein cholesterol, HDL-C; low-density lipoprotein cholesterol, LDL-C; systolic blood pressure, SBP.

## Data Availability

The original contributions presented in this study are included in the article. Further inquiries can be directed to the corresponding author.

## Abbreviations

ASCVD	Atherosclerotic cardiovascular disease
CKM	Cardiovascular–kidney–metabolic
CKD	Chronic kidney disease
NCDs	Chronic non-communicable diseases
DBP	Diastolic blood pressure
GFR	Glomerular filtration rate
HbA1c	Glycated hemoglobin
HDL-C	High-density lipoprotein cholesterol
LDL-C	Low-density lipoprotein cholesterol
SAH	Systemic arterial hypertension
SBP	Systolic blood pressure
T2DM	Type 2 diabetes mellitus
